# Association between facial expression and PTSD symptoms among young children exposed to the Great East Japan Earthquake: a pilot study

**DOI:** 10.3389/fpsyg.2015.01534

**Published:** 2015-10-13

**Authors:** Takeo Fujiwara, Rie Mizuki, Takahiro Miki, Claude Chemtob

**Affiliations:** ^1^Department of Social Medicine, National Research Institute for Child Health and DevelopmentTokyo, Japan; ^2^Department of Developmental Social Medicine, Mie University Graduate School/Faculty of MedicineTsu, Japan; ^3^Department of Psychosocial Medicine, Hospital of the National Center for Child Health and Development, National Research Institute for Child Health and DevelopmentTokyo, Japan; ^4^Department of Psychiatry, New York University School of MedicineNew York, NY, USA

**Keywords:** young children, facial emotion reactivity, facial expression, post-traumatic stress disorders, natural disaster, earthquakes

## Abstract

“Emotional numbing” is a symptom of post-traumatic stress disorder (PTSD) characterized by a loss of interest in usually enjoyable activities, feeling detached from others, and an inability to express a full range of emotions. Emotional numbing is usually assessed through self-report, and is particularly difficult to ascertain among young children. We conducted a pilot study to explore the use of facial expression ratings in response to a comedy video clip to assess emotional reactivity among preschool children directly exposed to the Great East Japan Earthquake. This study included 23 child participants. Child PTSD symptoms were measured using a modified version of the Parent’s Report of the Child’s Reaction to Stress scale. Children were filmed while watching a 2-min video compilation of natural scenes (‘baseline video’) followed by a 2-min video clip from a television comedy (‘comedy video’). Children’s facial expressions were processed the using Noldus FaceReader software, which implements the Facial Action Coding System (FACS). We investigated the association between PTSD symptom scores and facial emotion reactivity using linear regression analysis. Children with higher PTSD symptom scores showed a significantly greater proportion of neutral facial expressions, controlling for sex, age, and baseline facial expression (*p* < 0.05). This pilot study suggests that facial emotion reactivity, measured using facial expression recognition software, has the potential to index emotional numbing in young children. This pilot study adds to the emerging literature on using experimental psychopathology methods to characterize children’s reactions to disasters.

## Introduction

Children who experience severe trauma are more likely to later develop emotional numbing (Nugent et al., 2006; Kerig et al., 2012). The 4th edition of the Diagnostic and Statistical Manual (DSM) defines emotional numbing as having a “markedly diminished interest in significant activities… feelings of detachment or estrangement from others, and … restricted range of affect” (American Psychiatric Association, 1994). Emotional numbing is usually evaluated through self-reported questionnaires; however, it remains a challenge to assess the condition among young children because of their language limitations.

Kerig et al. (2012) revealed that trauma experience was significantly associated with a general numbing of emotion, which induced callousness among adolescents in juvenile detention centers. Further, it was reported that emotional numbing symptoms in women with post-traumatic stress disorder (PTSD) were associated with less positive affect in response to positive stimulus, and with a reduced blood oxygenation level-dependent response within the dorsomedial prefrontal cortex during positive stimulus (Frewen et al., 2012). Previous studies have indicated that the degree of emotional numbing can be predicted by PTSD symptom severity (Ehlers and Clark, 2000; Eftekhari et al., 2009; Ehring and Quack, 2010), and Litz (1992) has suggested that facial emotion reactivity could be a useful index to measure the degree of emotional numbing.

Prior research focusing on facial expressiveness has relied on human observers rating facial expressions recorded on video (Haynie and Lamb, 1995; Spielman et al., 2003). This is a very time-consuming and therefore expensive process. Recently, new technologies to capture facial emotional expression have been rapidly developed. Noldus FaceReader software automatically analyzes facial expressiveness (den Uyl and van Kuilenberg, 2005; Truong et al., 2008), and has been validated in a previous study (e.g., happiness emotion, *r* = 0.61) (Lewinski et al., 2014). Thus, we hypothesized that facial emotional expression can be a good marker of emotional numbing among young children who have been exposed to severe trauma, and new technologies to capture facial emotional expression can be used to detect young children with PTSD symptoms. This study explored whether facial emotion reactivity could be measured using this new technology among young children exposed to the Great East Japan Earthquake.

## Materials and Methods

### Ethical Statement

The Research Ethics Committee at the National Center for Child Health and Development approved this study (reference number: 714). Informed consent was obtained from the caregivers of all child participants. Further, we obtained informal assent from participating children.

### Participants

As part of a larger study to investigate the impact of the 2011 Great East Japan Earthquake on young children (Fujiwara et al., 2014), a convenience sample of children aged 4–6 years old at the time of the earthquake was recruited from two preschools in a coastal city located approximately 150 km from the epicenter. Children were recruited 18 months after the disaster and were aged 6–8 years old when they participated in this study on facial emotional reactivity. The school principal and staff of each preschool informed the children’s caregivers about the study (*N* = 60). A large proportion of families (93.3%) participated in the larger study. Although 35 children initially participated in the facial emotional expression study, only 23 records were useable for both the ‘baseline’ and the ‘comedy’ video clips. PTSD symptom severity did not differ between the participants whose records were useable and those whose were not (*p* = 0.75).

### Exposure

Child psychiatrists or clinical psychologists interviewed all participating children to obtain information about whether children had experienced one or more of the following traumatic events: the death of an immediate family member, a relative, or friend; seeing dead bodies; separation from parents, and witnessing tsunami waves or fire. Information about damage to the home (complete or partial), living in shelters, and living in temporary quarters or relatives’ homes was provided by caregivers using a self-report questionnaire. Exposure was coded as dichotomous (“yes” or “no”). A total exposure index was created by adding up exposure items in order to assess the severity of trauma.

### PTSD Symptoms

Post-traumatic stress disorder symptom severity was measured using the 28-item Japanese version of the Parent’s Report of the Child’s Reaction to Stress scale (Fletcher, 1996). Responses were rated on a 6-point Likert scale. The total score was distributed normally and reliability was high (Cronbach’s alpha = 0.86). The total PTSD symptoms score was used as a continuous variable.

### Facial Expressions

**FIGURE 1:**
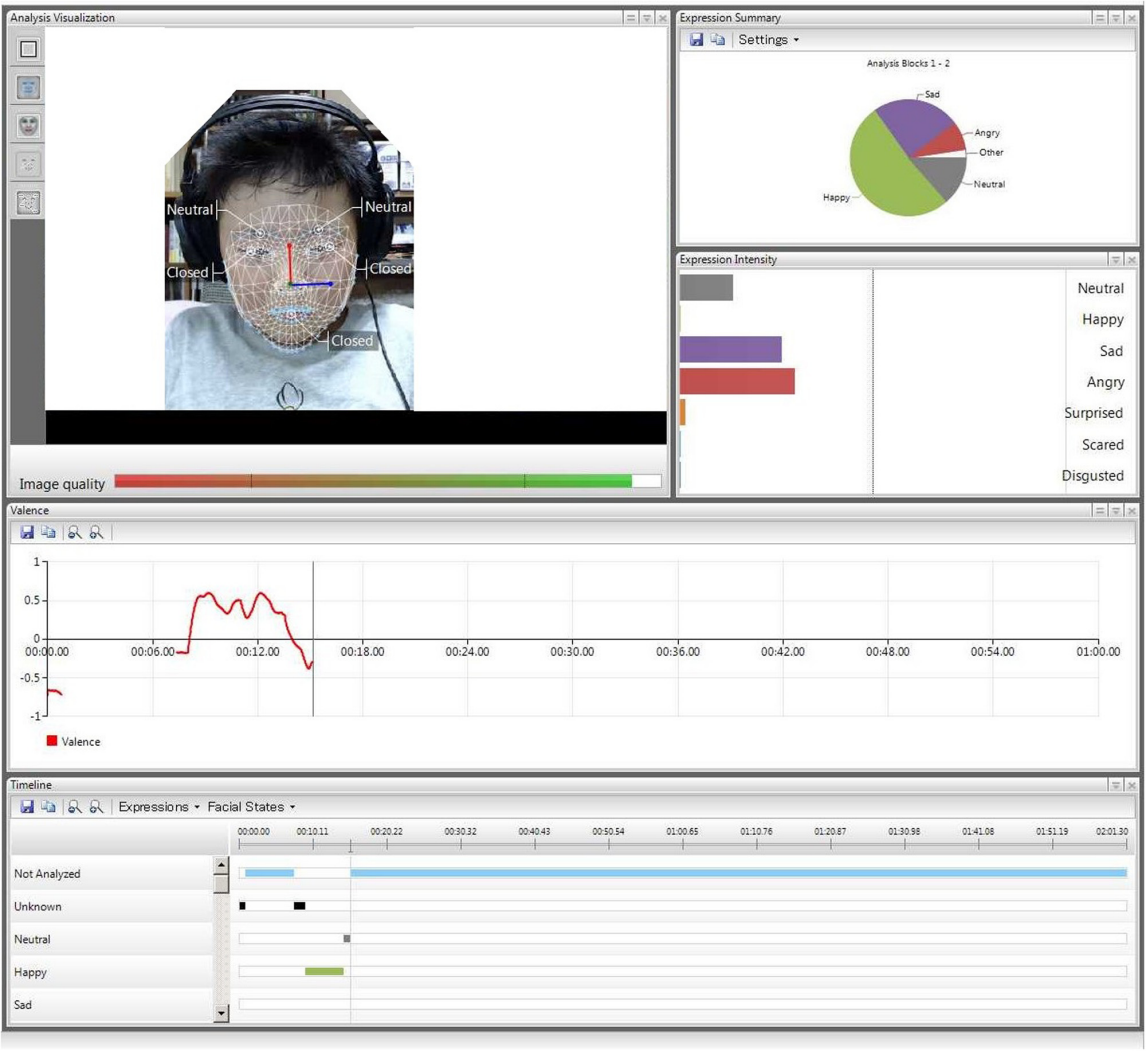
**Image of FaceReader software coding facial expression**.

The proportion of each facial expression (happy, sad, angry, surprised, scared, disgusted, and neutral) while viewing the ‘comedy’ video clip was measured using the Noldus FaceReader software for automatic facial expression analysis (den Uyl and van Kuilenberg, 2005; Truong et al., 2008) in 2013, around 3 years after the earthquake (see **Figure 1** for image). This software significantly reduces the burden for behavioral coding, keeping the quality of behavioral data (Chentsova-Dutton and Tsai, 2010). FaceReader identifies emotions expressed using the Facial Action Coding System (FACS; Ekman et al., 2002), and uses 55 key locations on the face to rate emotional expressions. FaceReader outputs have high convergent validity (95.9%) with FACS expert ratings (den Uyl and van Kuilenberg, 2005; Loijens and Krips, 2008). Although FaceReader software was primarily validated using adult faces, we confirmed its accuracy by utilizing it with children who had not been exposed to trauma (*n* = 9). After watching the “comedy” video clip, self-reported ratings of amusement (Likert scale, range 0–5) were marginally inversely associated with neutral facial expressions (*r* = –0.94, *p* = 0.063). Further, self-reported feelings of happiness were significantly positively associated with happy facial expressions (*r* = 0.98, *p* = 0.017), as were self-reported ratings of feeling sad with sad facial expressions (*r* = 0.96, *p* = 0.040). However, among trauma-exposed children (*n* = 25), self-rated emotions, and facial expressions were not correlated (neutral, *r* = 0.16; happy, *r* = –0.27; sad, *r* = 0.03, all *p* > 0.2). We recognize that further validation of the FaceReader is desirable.

### Procedures

Children watched a 2-min video clip showing a compilation of scenes from nature to establish a baseline for emotional reactivity (‘baseline’ video clip). The compilation of scenes was created using several images of the sky, clouds, mountains, waterfalls, and fields of flowers. The images were carefully selected so that they did not remind children of the earthquake (images of waves or the ocean were avoided). The video was shown on a laptop computer with a 15.6 inch screen. A video camera on top of the laptop’s display was used to capture facial expressions of the participants. Children then viewed a 2-min video clip from “The Library”, an episode of the TV comedy *Mr. Bean* (‘comedy’ video clip). Children’s facial expressions in reaction to the ‘baseline’ and ‘comedy’ video clips were coded using FaceReader software. The proportion of each facial expression was calculated based on the duration of each facial expression type, divided by the total duration. We calculated the proportion of each facial expression by dividing the time that the facial expression occurred by the total number of minutes, to adjust for individual differences in facial expression for both the ‘baseline’ and ‘comedy’ video clips. This approach controlled for individual differences in baseline facial expression. To determine whether Japanese children regarded *Mr. Bean* to be funny, self-reported ratings of amusement were assessed using a Likert scale ranging from 0 to 5. This assessment confirmed that children reported more amusement after watching *Mr. Bean* than after watching the ‘baseline’ clip (3.91 vs. 3.09, *p* = 0.023). Self-reported ratings of amusement were not associated with PTSD symptom scores (coefficient: –0.03, *t* = –1.15, *p* = 0.26).

### Data Analysis

We investigated the association between the proportion of each facial expression type after watching the ‘comedy’ video clip, and the total score from the Parent’s Report of the Child’s Reaction to Stress scale (PTSD symptom scores), which was adjusted for age, sex, and baseline proportion of the investigated type of facial emotion using linear regression. We focused primarily on the most frequently used facial expressions (neutral, sad, and happy).

## Results

The mean age of participants was 7.2 years (*SD* = 1.1) (**Table 1**). The sex of participants was almost equally distributed. PTSD symptom scores were distributed normally, with a mean score of 47.7 out of a possible 140, and ranged from 29 to 86 (*SD* = 13.2). PTSD symptom scores were not associated with the number of trauma experiences in this sample (*p* = 0.38), thus number of trauma experiences was not used as a covariate.

**Table 1:** Characteristics of sample (*N* = 23).

		Total	
		*M* or *N*	*SD* or %
Age	Year	7.2	1.1
Sex	Male	12	52.2
	Female	11	47.8
PTSD symptom scores	Range: 28–140	47.7	13.2
Number of trauma experiences		3.5	2.0

**Table 2** shows the proportion of each emotion. For both the ‘baseline’ and ‘comedy’ video clips, the largest mean proportion of facial expressions was for neutral faces. On average, around 40% of facial expressions were rated as neutral, ranging from 16 to 76% (*SD* = 17%) for the ‘baseline’ video, and 0.3 to 96% (*SD* = 32%) for the ‘comedy’ video, respectively. The mean proportion of each facial expression was as follows: sad, 36 and 25%; happy, 10 and 22%, for the ‘baseline’ and ‘comedy’ videos, respectively. These three facial expressions represented the largest proportion of facial expression in the study. We focused on neutral, happy, and sad facial expressions because they were most widely represented in the responses to the ‘comedy’ video. There were no gender differences in facial emotion expressiveness (data not shown).

**Table 2:** Proportion of facial expressions for baseline and comedy video.

	Type of facial expression	‘Baseline’ video		‘Comedy’ video	
		*M*	*SD*	*M*	*SD*
Proportion of facial expressions	Neutral	0.39	0.17	0.38	0.31


	Happy	0.10	0.14	0.24	0.26
	Sad	0.36	0.20	0.25	0.24
	Angry	0.11	0.11	0.13	0.19
	Surprised	0.03	0.07	0.005	0.007
	Scared	0.007	0.018	0.005	0.017
	Disgusted	0.003	0.004	0.004	0.007

We then conducted multivariate linear regression analysis to investigate the association between PTSD symptom scores and the proportion of neutral, happy, and sad facial expressions, controlling for age, sex, and baseline for each facial expression (**Table 3**). PTSD symptom scores were positively significantly associated with the proportion of neutral facial expressions observed during the ‘comedy’ video, after adjustment for age, sex, and baseline neutral facial expression [β = 0.01, *t*(22) = 2.11, *p* = 0.049]. Further, PTSD symptom scores were inversely significantly associated with the proportion of sad facial expressions, suggesting that children who showed higher PTSD symptom scores had less sad facial expressiveness [β = –0.01, *t*(22) = –2.65, *p* = 0.016]. There were no differences between groups regarding the proportion of happy facial expressions [β = 0.002, *t*(22) = 0.45, *p* = 0.66].

**Table 3:** Association between Facial Expression and PTSD Symptoms Score^a^.

		Neutral	Happy	Sad
PTSD symptoms score		0.01^∗^	0.002	–0.01^∗^
Age (unit: years)		–0.06	0.007	0.04
Sex (reference: male)		0.02	–0.16	0.04
Proportion of baseline facial expression	Neutral	0.70^∗^		
	Happy		0.76	
	Sad			0.29

*Post-traumatic stress disorder symptoms score was the total score of the Japanese short-version of The Parent’s Report of the Child’s Reaction to Stress scale.*

*^a^Coefficients for each facial expression adjusted for age, sex, and baseline proportion of each facial expression were shown.*

*^∗^p < 0.05.*

## Discussion

We found that neutral facial expressions shown by participants during the ‘comedy’ video were positively associated with PTSD symptoms in young children. In addition, there was a significant trend indicating that children with higher PTSD symptom scores were less likely to display sad facial expressions while watching the ‘comedy’ video. Our findings add to the literature that emotional numbing due to trauma experience measured by facial expression recognition technology can be predicted by PTSD symptoms; in contrast, emotional numbing was measured by human raters in previous studies (Ehlers and Clark, 2000; Eftekhari et al., 2009; Ehring and Quack, 2010).

Facial expressions were classified as neutral at a 40% rate of reaction to both the ‘baseline’ and ‘comedy’ videos. Happy facial expressions increased from 10 to 24%, and sad facial expressions were reduced from 36 to 25% when watching the “comedy” video. These results suggest that the ‘comedy’ video was effective as a means of provoking emotional reactions from the participating children. Children with higher PTSD symptom scores were significantly more likely to display a neutral facial expression, and were less likely to display a sad facial expression. These findings support Litz’s (1992) hypothesis that high PTSD symptom scores are more likely to be associated with a greater restriction in affective reaction and expression to emotional stimuli. Alternatively, children with higher PTSD symptom scores might be unable to respond to outside stimulus. However, the reason for no differences in the proportion of happy expressions between groups is uncertain.

In addition, this study provides preliminary evidence that suggests the feasibility of using automated facial expression detection technology to measure emotional responsiveness. This technology has potential applications in research involving PTSD among both children and adults. Previous studies using emotional facial expression software were limited to adults (Samal and Iyengar, 1992; Zeng et al., 2009; Olderbak et al., 2014). Prior studies have not used the software with child study participants. Thus, to the best of our knowledge, this is the first study to suggest the possible utility of this software for children. For example, this technology may be useful in characterizing emotional responsiveness among children or adults with limited language skills.

A number of limitations need to be addressed. First, our small sample size precludes a firm conclusion. Second, PTSD symptoms were not validated among Japanese children. Third, not all participating children may have considered the TV comedy show used in the ‘comedy’ video clip to be funny; therefore, the clip cannot be generalized as funny stimuli. Fourth, facial expressions from natural stimulus, such as parental positive involvement, may differ from expressions from responses to the artificial stimulus used in this study.

Future research should include a larger number of participants who have a wider range of PTSD symptoms. The finding that children with higher PTSD symptom scores had restricted facial expressiveness of sad emotions is somewhat paradoxical, because it would be expected that children with a greater severity of PTSD would use sad expressions more often as a means to alert caregivers to a greater need for support. This finding should be explored in adults as well as children in both the post-disaster context and following exposure to other types of trauma. Future research should investigate whether interpersonal signaling of distress is impaired among trauma-exposed children. If this finding were to be confirmed, it would be potentially important in guiding case detection of children with PTSD symptoms in the aftermath of large-scale trauma exposure, such as natural disasters. Further development and use of technology to assess facial reactivity would provide a promising tool for researchers and clinicians to explore.

## Conflict of Interest Statement

The authors declare that the research was conducted in the absence of any commercial or financial relationships that could be construed as a potential conflict of interest.

## References

[B1] American Psychiatric Association (1994). *The Diagnostic and Statistical Manual of Mental Disorders*, 4th Edn. Washington, DC: American Psychiatric Association.

[B2] Chentsova-Dutton Y. E. Tsai J. L. (2010). Self-focused attention and emotional reactivity: the role of culture. *J. Pers. Soc. Psychol.* 98 507–519. 10.1037/a0018534 20175627

[B3] den Uyl M. van Kuilenberg H. (2005). “The FaceReader: online facial expression recognition,” in *Proceedings of Measuring Behavior 2005, 5th International Conference on Methods and Techniques in Behavioral Research*, eds Noldus L. P. J. J. Grieco F. Loijens L. W. S. Zimmerman P. H. (Wageningen: Noldus Information Technology), 589–590.

[B4] Eftekhari A. Zoellner L. A. Vigil S. A. (2009). Patterns of emotion regulation and psychopathology. *Anxiety Stress Coping* 22 571–586. 10.1080/10615800802179860 19381989 PMC3234115

[B5] Ehlers A. Clark D. M. (2000). A cognitive model of posttraumatic stress disorder. *Behav. Res. Ther.* 38 319–345. 10.1016/S0005-7967(99)00123-0 10761279

[B6] Ehring T. Quack D. (2010). Emotion regulation difficulties in trauma survivors: the role of trauma type and PTSD symptom severity. *Behav. Ther.* 41 587–598. 10.1016/j.beth.2010.04.004 21035621

[B7] Ekman P. Friesen W. V. Hager J. C. (2002). *The Facial Action Coding System*, 2nd Edn. Salt Lake City, UT: Research Nexus eBook.

[B8] Fletcher K. (1996). “Psychometric review of the Parent Report of Child’s Reaction to Stress,” in *Measurement of Stress, Trauma, and Adaptation*, ed. Stamm B. H. (Lutherville, MD: Sidran Press), 225–227.

[B9] Frewen P. A. Dozois D. J. Neufeld R. W. Lane R. D. Densmore M. Stevens T. K. (2012). Emotional numbing in posttraumatic stress disorder: a functional magnetic resonance imaging study. *J. Clin. Psychiatry* 73 431–436. 10.4088/JCP.10m06477 22154897

[B10] Fujiwara T. Yagi J. Homma H. Mashiko H. Nagao K. Okuyama M. (2014). Clinically significant behavior problems among young children 2 years after the Great East Japan Earthquake. *PLoS ONE* 9:e109342 10.1371/journal.pone.0109342 PMC4204852 25333762

[B11] Haynie D. L. Lamb M. E. (1995). Positive and negative facial expressiveness in 7-, 10-, and 13-month-old infants. *Infant Behav. Dev.* 18 257–259. 10.1016/0163-6383(95)90055-1

[B12] Kerig P. K. Bennett D. C. Thompson M. Becker S. P. (2012). “Nothing really matters”: emotional numbing as a link between trauma exposure and callousness in delinquent youth. *J. Trauma Stress* 25 272–279. 10.1002/jts.21700 22615202

[B13] Lewinski P. Fransen M. L. Tan E. (2014). Predicting advertising effectiveness by facial expressions in response to amusing persuasive stimuli. *J. Neurosci. Psychol. Econ.* 7 1–14. 10.1037/npe0000012

[B14] Litz B. (1992). Emotional numbing in combat-related post-traumatic stress disorder: a critical review and reformulation. *Clin. Psychol. Rev* 12 417–432. 10.1016/0272-7358(92)90125-R

[B15] Loijens L. Krips O. (2008). *FaceReader Methodology.* Available at: http://www.noldus.com/webfm_send/618

[B16] Nugent N. R. Christopher N. C. Delahanty D. L. (2006). Initial physiological responses and perceived hyperarousal predict subsequent emotional numbing in pediatric injury patients. *J. Trauma Stress* 19 349–359. 10.1002/jts.20130 16789001

[B17] Olderbak S. Hildebrandt A. Pinkpank T. Sommer W. Wilhelm O. (2014). Psychometric challenges and proposed solutions when scoring facial emotion expression codes. *Behav. Res. Methods* 46 992–1006. 10.3758/s13428-013-0421-3 24311061 PMC4237926

[B18] Samal A. Iyengar P. A. (1992). Automatic recognition and analysis of human faces and facial expressions:a survey. *Pattern Recognit.* 25 65–77. 10.1016/0031-3203(92)90007-6

[B19] Spielman J. L. Borod J. C. Ramig L. O. (2003). The effects of intensive voice treatment on facial expressiveness in Parkinson disease: preliminary data. *Cogn. Behav. Neurol.* 16 177–188. 10.1097/00146965-200309000-00005 14501539

[B20] Truong K. P. Neerincx M. A. van Leeuwen D. A. (2008). “Measuring spontaneous vocal and facial emotion expressions in real world environments,” in *Proceedings of Measuring Behavior 2008*, eds Spink A. J. Ballintijn M. R. Bogers N. D. Grieco F. Loijens L. W. S. Noldus L. P. J. J. (Maastricht: Noldus Information Technology), 170–171.

[B21] Zeng Z. Pantic M. Roisman G. I. Huang T. S. (2009). A survey of affect recognition methods: audio, visual, and spontaneous expressions. *IEEE Trans. Pattern Anal. Mach. Intell.* 31 39–58. 10.1109/TPAMI.2008.52 19029545

